# Mapping Polycomb Response Elements at the *Drosophila melanogaster giant* Locus

**DOI:** 10.1534/g3.113.008896

**Published:** 2013-10-29

**Authors:** Jumana AlHaj Abed, Connie L. Cheng, Chase R. Crowell, Laura L. Madigan, Erica Onwuegbuchu, Siddhi Desai, Judith Benes, Richard S. Jones

**Affiliations:** Department of Biological Sciences, Dedman Life Sciences Building, Southern Methodist University, Dallas, Texas 75275-0376

**Keywords:** polycomb, polycomb response element, gene silencing, pairing-sensitive silencing, epigenetic

## Abstract

Polycomb-group (PcG) proteins are highly conserved epigenetic transcriptional regulators. They are capable of either maintaining the transcriptional silence of target genes through many cell cycles or enabling a dynamic regulation of gene expression in stem cells. In *Drosophila melanogaster*, recruitment of PcG proteins to targets requires the presence of at least one polycomb response element (PRE). Although the sequence requirements for PREs are not well-defined, the presence of Pho, a PRE-binding PcG protein, is a very good PRE indicator. In this study, we identify two PRE-containing regions at the PcG target gene, *giant*, one at the promoter, and another approximately 6 kb upstream. PRE-containing fragments, which coincide with localized presence of Pho in chromatin immunoprecipitations, were shown to maintain restricted expression of a *lac*Z reporter gene in embryos and to cause pairing-sensitive silencing of the mini-*white* gene in eyes. Our results also reinforce previous observations that although PRE maintenance and pairing-sensitive silencing activities are closely linked, the sequence requirements for these functions are not identical.

Polycomb-group (PcG) genes were initially identified as repressors of Hox genes in *Drosophila melanogaster* (Lewis 1978). In recent years, the protein products of these epigenetic regulators have been shown to be localized at hundreds of chromosomal sites (Negre *et al.* 2006; Schwartz *et al.* 2006; Tolhuis *et al.* 2006; Oktaba *et al.* 2008). The majority of their target genes are regulators of development, cell-cycle progression, and/or cell signaling (Oktaba *et al.* 2008; Classen *et al.* 2009; Martinez *et al.* 2009). In PcG mutants, target genes are expressed outside their usual domains of expression, leading to defects in body development. The most conspicuous examples are homeotic transformations that are caused by ectopic expression of Hox genes (Struhl 1983; Wedeen *et al.* 1986; Simon *et al.* 1992). Mammalian PcG proteins are required to maintain the pluripotent state of stem cells and their misexpression contributes to a wide variety of human cancers (Richly *et al.* 2011).

Three major *Drosophila* PcG protein complexes have been described, Polycomb repressive complex 1 (PRC1), PRC2, and Pleiohomeotic (Pho)-RC. Additional *Drosophila* complexes that contain PcG proteins dRAF and PR-DUB also have been identified (Lagarou *et al.* 2008; Scheuermann *et al.* 2010). Simon and Kingston (2013) recently presented a thorough review of PcG complexes and their activities. Pho-RC consists of the sequence-specific DNA binding protein Pho and Scm-related gene containing four mbt domains (dSfmbt) (Klymenko *et al.* 2006). The core components of PRC1 are Polycomb (Pc), Polyhomeotic (Ph), Posterior sex combs (Psc), and Sex combs extra (Sce, also known as dRing) (Shao *et al.* 1999; Saurin *et al.* 2001). The core components of PRC2 are Enhancer of zeste [E(z)], Extra sex combs (Esc), Suppressor of zeste 12 [Su(z)12], and NURF55 (Czermin *et al.* 2002; Müller *et al.* 2002). Multiple variants of Drosophila and mammalian PRC1 and PRC2 complexes have been identified with alternative subunit compositions, which may confer distinct biochemical activities (Simon and Kingston 2013).

Based on examination of the interdependencies of components of Pho-RC, PRC1, and PRC2 for target site binding, a hierarchical binding pathway has been proposed (Wang *et al.* 2004b). After DNA binding by Pho, PRC2 is recruited by direct interaction with Pho. The E(z) subunit of PRC2 then trimethylates histone H3 at lysine 27 (H3K27me3), facilitating binding by PRC1 because of the affinity of the Pc chromo domain for H3K27me3 (Fischle *et al.* 2003; Min *et al.* 2003). However, it is likely that PRC2-independent PRC1 recruitment pathways also exist at some loci (Schwartz *et al.* 2006). PRC1 may contribute to transcriptional repression by a variety of mechanisms that include monoubiquitylation of histone H2A (H2Aub1), localized chromatin compaction, and/or inhibitory interactions with transcription machinery (Francis *et al.* 2004; Wang *et al.* 2004a; Lehmann *et al.* 2012).

In *Drosophila*, recruitment of PcG proteins to specific chromosomal sites and repression of nearby genes require the presence of one or more polycomb response elements (PREs) (Simon *et al.* 1993). Although PcG proteins have been shown to be present at hundreds of genomic locations, PREs at fewer than 20 genes have been functionally characterized. A number of DNA-binding factors have been identified that may contribute to PRE function, including Pho (Brown *et al.* 1998; Fritsch *et al.* 1999), Pleiohomeotic-like (Phol) (Brown *et al.* 2003), GAGA factor (Gaf) (Horard *et al.* 2000; Mahmoudi *et al.* 2003), Pipsqueak (Psq) (Lehmann *et al.* 1998), Zeste (z) (Dejardin and Cavalli 2004), Sp1/Kruppel-like factor (Spps) (Brown *et al.* 2005; Brown and Kassis 2010), Dorsal switch protein 1 (Dsp1) (Dejardin *et al.* 2005), and Grainyhead (Grh), (Blastyak *et al.* 2006), but the exact sequence requirements for PRE activity remain elusive (Kassis and Brown 2013). This is attributable to the heterogeneous sequence organization of PREs and low conservation of consensus sequences for the binding sites of PRE-binding proteins. Among the PRE-binding proteins, only Pho has been detected at all characterized PREs (Kassis and Brown 2013). The other factors appear to be present in various combinations at different PREs. Therefore, Pho localization is a very good indicator of the presence of a biologically functional PRE.

Although the locations of PREs predicted by bioinformatics approaches have shown a low correlation with the distribution of PcG proteins in ChIP-on-chip studies (Ringrose *et al.* 2003; Fielder and Rehmsmeier 2006; Schwartz *et al.* 2006; Oktaba *et al.* 2008), PREs can be functionally defined by their ability to regulate expression of a reporter gene within the context of a transgene. In the PRE maintenance assay, DNA fragments are tested for their abilities to limit enhancer-driven expression of a reporter gene to the normal expression domains of the endogenous gene of the enhancer. This approach has been possible mainly because PREs have been shown to be able to regulate activities of heterologous promoters and enhancers. For example, in the absence of a PRE, enhancers from genes such as *Ultrabithorax* (*Ubx*) or *engrailed* (*en*) produce ectopic expression of a *lac*Z reporter, in addition to expression within the normal domains of *Ubx* and *en*, respectively. Inclusion of a PRE within the transgene prevents ectopic expression and maintains *lac*Z expression within the normal boundaries of the endogenous gene (Busturia *et al.* 1997; Americo *et al.* 2002; Cunningham *et al.* 2010; Park *et al.* 2012).

PREs also are able to repress expression of the mini-*white* reporter gene, resulting in lighter eye color. Furthermore, the majority of PREs produce a phenomenon known as pairing-sensitive silencing (Kassis *et al.* 1991). Normally, the eye pigmentation of flies with two copies of a mini-*white*–containing transgene is approximately twice that of flies with only one copy of the transgene. Pairing-sensitive silencing is observed when flies that are homozygous for the transgene have lighter eye colors compared to heterozygotes. However, not all DNA fragments that function as PREs in maintenance assays also produce pairing-sensitive silencing. For example, the *Mcp* core PRE requires additional sequences to act as a pairing-sensitive silencer (Muller *et al.* 1999).

*giant (gt)* is a zygotic gap gene that affects the development of the head and abdominal regions in *Drosophila.* Its identification as a PcG target gene was based on the isolation of dominant *E(z)* alleles that suppress the *nanos* (*nos*) maternal effect (Pelegri and Lehmann 1994). The Su(*nos*) phenotype was shown to be attributable to failure to maintain repression of the gap genes *gt* and *knirps* initiated by maternal Hunchback (Hb). ChIP-on-chip studies have confirmed the presence of PcG proteins at *gt* in embryos (Negre *et al.* 2006; Oktaba *et al.* 2008). Initially, *gt* is expressed in two broad stripes at the syncytial blastoderm stage. By the cellular blastoderm stage, the anterior stripe is divided into two stripes and a patch of expression at the anterior tip is observed (Eldon and Pirrotta 1991). This expression is controlled by four enhancers located upstream of the *gt* promoter (Berman *et al.* 2002; Schroeder *et al.* 2004): *gt*_(-3) (−1.3 to −2.5 kb) produces the posterior stripe; *gt*_(-10) (−8.8 to −10.5 kb) produces the major anterior stripes; *gt*_(-6) (−4.3 to −6.5 kb) produces the anterior tip expression; and *gt*_(-1) (−0.05 to −1.3 kb) produces both the posterior and major anterior stripes. The role played by the apparent redundancy of *gt*_(-1) with *gt*_(-10) and *gt*_(-3) in regulation of the endogenous *gt* gene is not clear. Beyond gastrulation, *gt* primarily is expressed in the head region (Eldon and Pirrotta 1991). Through the cellular blastoderm stage, it appears that PcG repression of *gt* is redundant with repression by gene-specific transcription factors, such as Hb (Pelegri and Lehmann 1994). However, *Pc* mutants exhibit ectopic *gt* expression in later embryonic stages (Negre *et al.* 2006). To initiate a more detailed analysis of PcG regulation of *gt*, we have mapped the locations of *gt* PREs.

## Materials and Methods

### *Drosophila* stocks and generation of transgenic lines

Strains are described at the Bloomington Drosophila Stock Center web site (http://flystocks.bio.indiana.edu) unless otherwise specified. To produce the SD10 constructs, *gt* genomic fragments were PCR-amplified from CH322-101F2 or CH322-5H16 BAC clones (BACPAC resources: http://www.pacmanfly.org/) using primers that included FRT sequences and *Nsp*I sites (Supporting Information, Table S1). Amplified fragments were digested with *Nsp*I and ligated to the vector *Sph*I site located between the *en* enhancer and promoter (in the same orientation relative to the *en* promoter as in the *gt* promoter). SD10-*gt* constructs were injected into *w^1118^* embryos by Genetics Services (Sudbury, MA) or BestGene (Chino Hills, CA). Additional lines were obtained by transposon mobilization using P[Δ2-3] (Robertson *et al.* 1988). The *gt* fragments were deleted from transgenes by crossing SD10-*gt* females to hsFLP males and heat-shocking embryos or first instar larvae for 1 hr at 37°. Deletions were verified by PCR using SD10C-U (5′GTTGAGCCGAAGAGAAAATACGC-3′) and SD10CL (5′-GTTTTCCCACTCACGACGTTG-3′) primers. To examine β-galactosidase expression in a PcG-mutant background, SD10-*gt* males were crossed to *ph-d^401^ ph-p^602^ w^1^/FM7c* females. To test for pairing-sensitive silencing, males were aged 2 days after eclosure and the eye colors of flies that were homozygous for the transgene were compared to those of flies that were heterozygous for the same transgene.

### Immunostaining of embryos

Embryos were collected at 25° then were fixed and processed essentially as previously described (Jones and Gelbart 1990) with the following modifications. Rabbit anti-β-galactosidase antibodies (Cappel) were diluted 1:1500. Biotin-SP–conjugated goat-anti-rabbit secondary antibodies (Jackson Immunoresearch) were diluted 1:10,000. Streptavidin-horseradish peroxidase (Jackson Immunoresearch) was diluted 1:5000. Signals were detected by incubating embryos in 1 mg/ml diaminobenzidine (Sigma-Aldrich) in 0.1 M Tris-HCl (pH 7), 1% NiCl_2_, and 0.003% H_2_O_2_ for approximately 20 min. Reactions were stopped by washing with PBST (0.01% Trition-X-100). Embryos were dehydrated and mounted in Permount (Fisher Scientific). Images were obtained using a Ziess Axiovert 200M microscope.

### Chromatin immunoprecipitation

The protocol was performed as indicated in the ChIP assay kit (Millipore) with the following modifications. *Oregon-R* embryos were collected for 30 min, aged for 170 min at 25°, and then dechorionated in 50% hypochlorite bleach, briefly washed with water, and fixed in 2% formaldehyde in PBS:heptane (1:3) for 20 min. Fixation was quenched by addition of glycine to a final concentration of 50 mM. Embryos were then washed, weighed, flash-frozen, and stored at −80°. Embryos were homogenized in 50 mM Tris (pH 8.1) and 10 mM EDTA supplemented with 1.25× complete EDTA-free protease inhibitor cocktail (Roche). SDS was added to a final concentration of 1%. After 10 min of incubation on ice, samples were sonicated in a cup horn sonicator (Misonix sonicator 3000) at 4° to produce DNA fragments that were predominantly within the range of 0.3 to 0.7 kb. An equivalent of 2 mg of embryos was set aside as input genomic DNA and incubated with 0.2 M NaCl overnight at 65° to reverse the crosslinks. The remainder of the supernatant was diluted 10-fold with ChIP dilution buffer, and chromatin from the equivalent of 3.2 mg of embryos was incubated with 2.5 μl rabbit anti-Pho antisera (Brown *et al.* 2003) or 5 μl rabbit pre-immune serum for mock.

### Quantitative PCR

Quantitative PCR was performed with PerfeCTa SYBR Green SuperMix (Quanta Biosciences) using a Rotor Gene RG3000 thermocycler (Corbett Research). Immunoprecipitated DNA from 100 μg of embryos was used for each PCR reaction. Sequences of primers are in Table S2. PCR reactions were performed in triplicate for each ChIP experiment. The data presented are the average of three independent ChIP experiments from independent chromatin preparations. Percent input values were calculated using Rotor Gene software by comparing Ct values of samples to standard curves established by the Ct values of total chromatin controls.

### Eye pigment assay

Eye pigmentation was quantified by homogenizing a total of 10 male heads (4 days after eclosion) from each fly group in 0.5 ml of 0.01 M HCl in ethanol. Homogenate was left overnight at 4°, warmed for 5 min at 50°, and then centrifuged. The absorbance of the supernatant was measured at 480 nm (Pal-Bhadra *et al.* 2004). Assays for each genotype were performed in triplicate. *P* values were calculated using unpaired *t* test to determine statistical significance.

## Results

### Pho binds to two locations within *gt cis*-regulatory region

**Figure 1:**
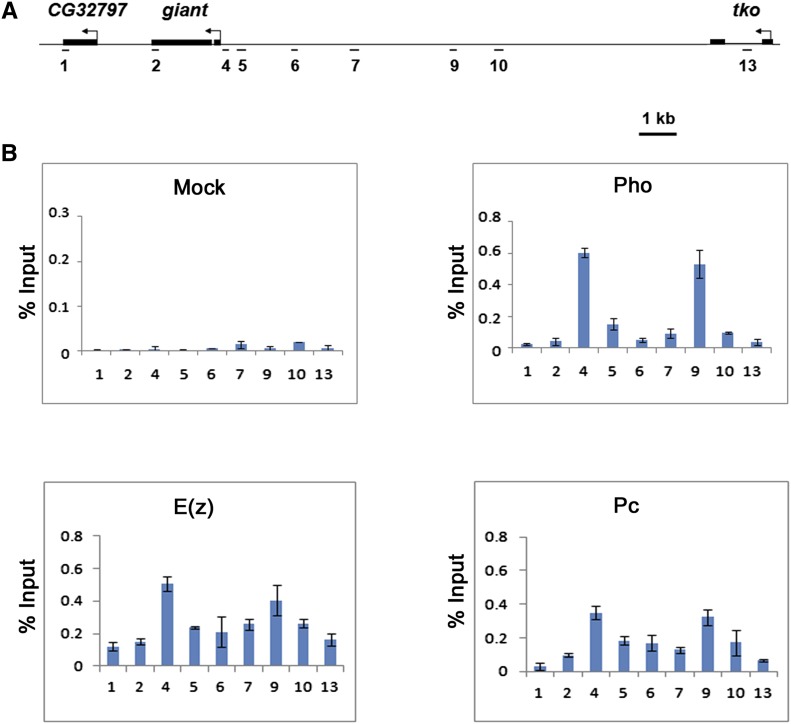
Pho binds to two regions within the *giant cis*-regulatory region that colocalizes with peaks of E(z) and Pc distribution. (A) Schematic representation showing the *giant* genomic region and flanking genes. Regions amplified by PCR in ChIP assays (1–13) are shown. Region 1 and 13 are, respectively, within the *CG32797* and *tko* genes and serve as negative controls. (B) ChIP analysis of *Oregon-R* embryos 2 h 50 min to 3 h 20 min after egg lay shows two Pho peaks, one close to the *gt* promoter (region 4) and the other ∼6 kb upstream (region 9). E(z) and Pc, subunits of PRC2 and PRC1, respectively, colocalize with Pho but are more broadly distributed. Preliminary ChIP assays using 33 primer sets spanning this 19-kb region did not show the presence of Pho at additional sites (data not shown). ChIP was performed as indicated with anti-Pho antibody (upper right panel), anti-E(z) antibody (lower left panel), anti-Pc antibody (lower right panel), and rabbit preimmune antiserum for mock (upper left panel). ChIP signals are presented as a percent of input chromatin. Error bars represent SD.

To date, all PREs that have been functionally tested bind Pho (Oktaba *et al.* 2008; Kassis and Brown 2013). Therefore, the presence of Pho at a given genomic region is a good indicator of the location of a biologically active PRE. To identify *gt* PREs, we began by precisely mapping the distribution of Pho across a 19-kb region that encompasses the *gt* locus and extends into flanking genes *CG32797* and *technical knockout* (*tko*) (Figure 1A). ChIP assays were performed on *Oregon-R* blastoderm stage embryos using anti-Pho antibodies. Pho was detected at two regions within the *gt* upstream regulatory region of *gt* (Figure 1B). The first is near the promoter at the PCR-amplified region 4 (+9 to −168). The second is approximately 6 kb upstream at region 9 (−6108 to −6320) (Figure 1). These results are consistent with a genome-wide ChIP-on-chip study that reported the presence of Pho at the *gt* locus in embryos (Oktaba *et al.* 2008). ChIP assays with anti-E(z) and anti-Pc antibodies show colocalization of these PRC2 and PRC1 subunits with Pho, but with broader distributions (Figure 1B). Even though the presence of Pho at these regions is highly suggestive that they contain PREs, *in vivo* tests, such as PRE maintenance or pairing-sensitive silencing assays, are required to confirm these predictions.

### *gt* has two PRE-containing regions that coincide with Pho localization

To test for PRE activity, *gt* genomic fragments were inserted into the SD10 *P*-element vector, which contains an *engrailed* (*en*) enhancer and promoter upstream of the *lac*Z reporter gene. The *en* enhancer produces *lac*Z expression in 14 stripes and resembles endogenous *en* expression. Without the presence of a PRE, ectopic expression of *lac*Z is detected between *en*-like stripes. However, the addition of an *en* PRE (Devido *et al.* 2008) or a heterologous PRE from the *invected* (*inv*) or *Ubx* (bxdd) genes (Cunningham *et al.* 2010) results in maintenance of the restricted *en*-like *lac*Z expression.

**Figure 2:**
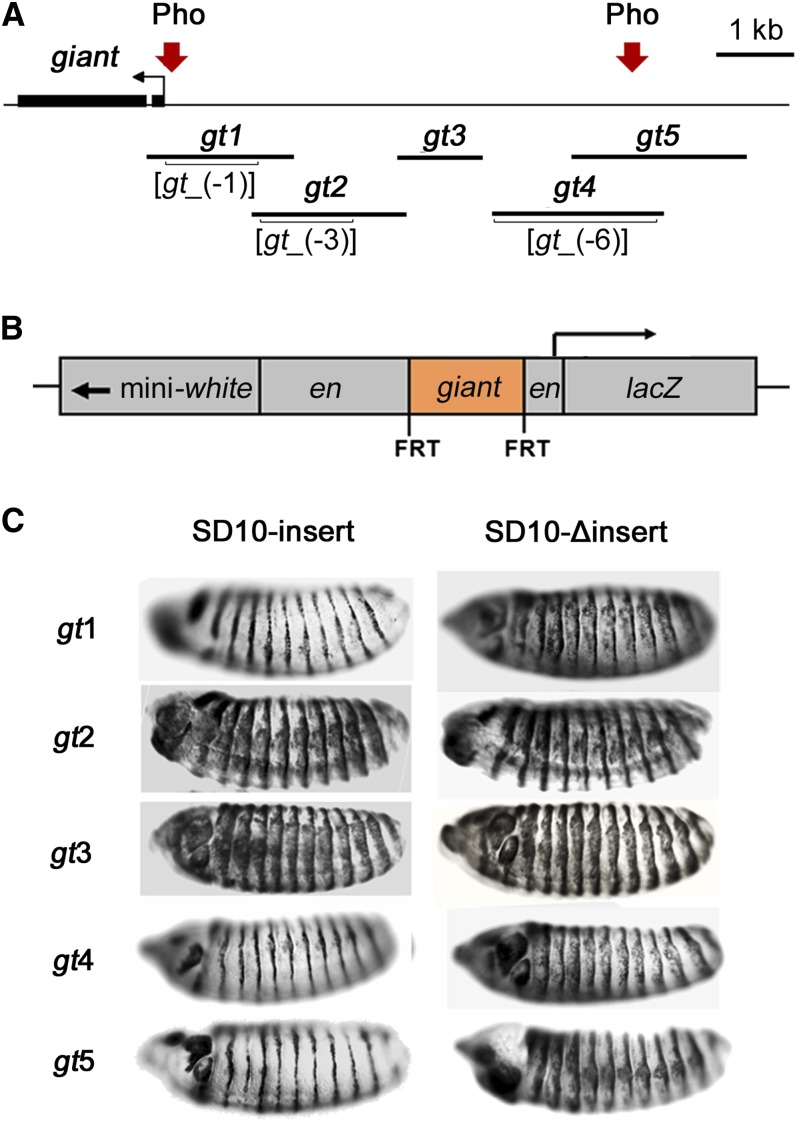
The *giant* inserts tested for their abilities to maintain *en*-like expression pattern of β-galactosidase. (A) A schematic of *gt* upstream regulatory region showing fragments *gt1–gt5* that were cloned into the SD10 vector and tested for PRE activity The locations of previously mapped *gt* enhancers *gt_(-1)*, *gt_(-3)*, and *gt_(-6)* are indicated in brackets. Pho-positive regions are indicated by arrows and correspond to PCR amplified regions 4 and 9 (Figure 1). (B) A schematic representation of the *en-lacZ* reporter construct SD10. Inserts are flanked by FRT sites. (C) Stage 14 embryos from transgenic lines stained for β-galactosidase expression. Lateral views of embryos are shown, anterior to the left, dorsal up. Transgenic lines tested are indicated to the left of the embryos. Expression patterns are representative of those produced by multiple lines of each construct. However, lines that failed to maintain the *en*-like pattern exhibited varying degrees of ectopic expression. Embryos containing intact transgenes are on the left. ΔInsert lines (right) are FLP recombinase–mediated deletion derivatives of the same lines shown on the left.

The *gt* fragments were inserted between the *en* upstream regulatory region and *en* promoter to produce SD10-*gt* constructs (Figure 2B). Each insert was flanked by FRT sites to allow precise excision of *gt* insert by FLP recombinase *in vivo*. By comparing reporter expression from the intact transgenes and insert-deleted derivatives, it is possible to distinguish regulatory effects of the *gt* insert from potential position effect of the genomic location of the transgene.

Guided by the locations of Pho-positive ChIP regions and the locations of *gt* embryonic enhancers (Berman *et al.* 2002; Schroeder *et al.* 2004), five overlapping fragments were selected for analysis (Figure 2A): *gt*1 includes the first Pho-positive region by ChIP, and the entire *gt*_(-1) enhancer; *gt*2 contains the entire *gt*_(-3) enhancer; *gt*3 does not include an identified enhancer and does not show Pho binding by ChIP; *gt*4 contains the *gt*_(-6) enhancer; and the *gt*4 and *gt*5 fragments overlap by 1.2 kb and contain the second Pho-positive region. The constructs were used to produce multiple transgenic lines. The inserts were deleted from a subset of these lines by FLP recombinase. Stage 14 embryos containing intact transgenes and their deletion derivatives were tested for β-galactosidase expression.

Embryos from each of the assayed transgenic lines containing SD10-*gt1*, SD10-*gt4*, or SD10-*gt5* showed restricted *en*-like patterns of β-galactosidase expression, indicative of the presence of a PRE within those constructs, whereas all SD10-*gt2* and SD10-*gt3* lines showed distinct ectopic expression of the *lac*Z reporter. Representative embryos for each construct are shown for each in Figure 2C. Maintenance of the *en*-like expression pattern was lost upon excision of *gt*1, *gt*4, and *gt*5 inserts (Figure 2C). Ectopic β-galactosidase expression was observed before and after the deletion of *gt*2 or *gt*3 inserts. A summary of all lines tested is shown in Table 1. Based on the results of these PRE maintenance assays, we conclude that the *gt*1, *gt*4, and *gt*5 fragments contain PREs. These results are consistent with the localization of Pho at region 4 within the *gt*1 fragment and at region 9 within the region of overlap between *gt*4 and *gt*5.

**Table 1:** Summary of PRE maintenance assay results

**Construct**	**Pho**	**PRE Function**
*gt1*	+	5/5
*gt2*	−	0/4
*gt3*	−	0/5
*gt4*	+	5/5
*gt5*	+	2/2

A list of SD10-*gt* constructs indicating whether Pho was found to bind to the respective *gt* fragments in ChIP assays and the number of lines exhibiting PRE activity in maintenance assays (PRE function).

### Maintenance of repression by *gt* fragments is PcG-dependent

**Figure 3:**
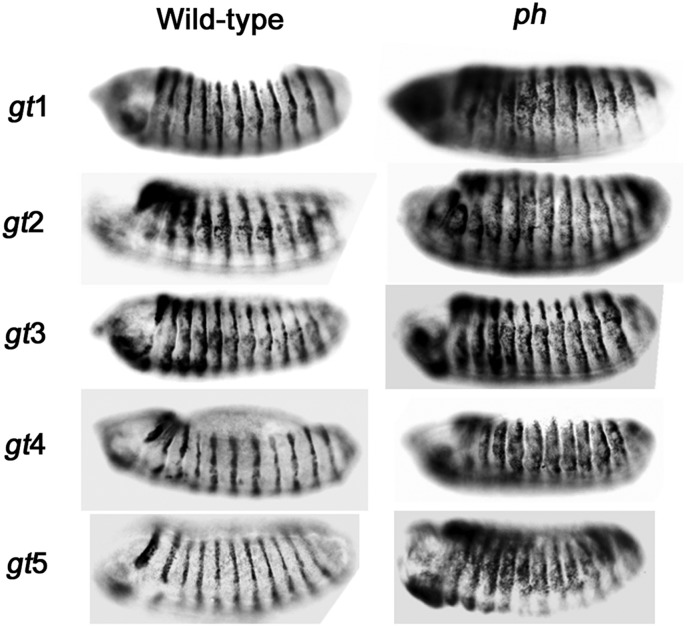
Maintenance of SD10-*gt* reporter repression is PcG-dependent. Stage 14 embryos from transgenic lines stained for β-galactosidase expression. Embryos with a wild-type PcG background are on the left. Embryos from crosses of transgenic lines to *ph-d^401^ ph-p^602^* double mutants are on the right. Orientation and identification of embryos are the same as in Figure 2C.

To confirm that the repressive ability of *gt* fragments is PcG-dependent, β-galactosidase expression was analyzed in a PcG mutant background. Representative transgenic lines for each construct were crossed to a *polyhomeotic* (*ph*) mutant (*ph-d^401^ ph-p^602^* double mutants) and β-galactosidase expression was compared to the same transgenic lines in a wild-type PcG background (Figure 3). SD10-*gt1*, SD10-*gt4*, and SD10-*gt5* all showed ectopic β-galactosidase expression in the *ph* mutant. This PcG-dependent maintenance of reporter gene repression confirms that SD10-*gt1*, SD10-*gt4*, and SD10-*gt5* contain PREs. SD10-*gt2* and SD10-*gt3* constructs showed ectopic β-galactosidase expression in both *ph^+^* and *ph^−^* backgrounds, consistent with lack of a functional PRE in these fragments.

### The *gt* fragments are able to demonstrate pairing-sensitive silencing

**Figure 4:**
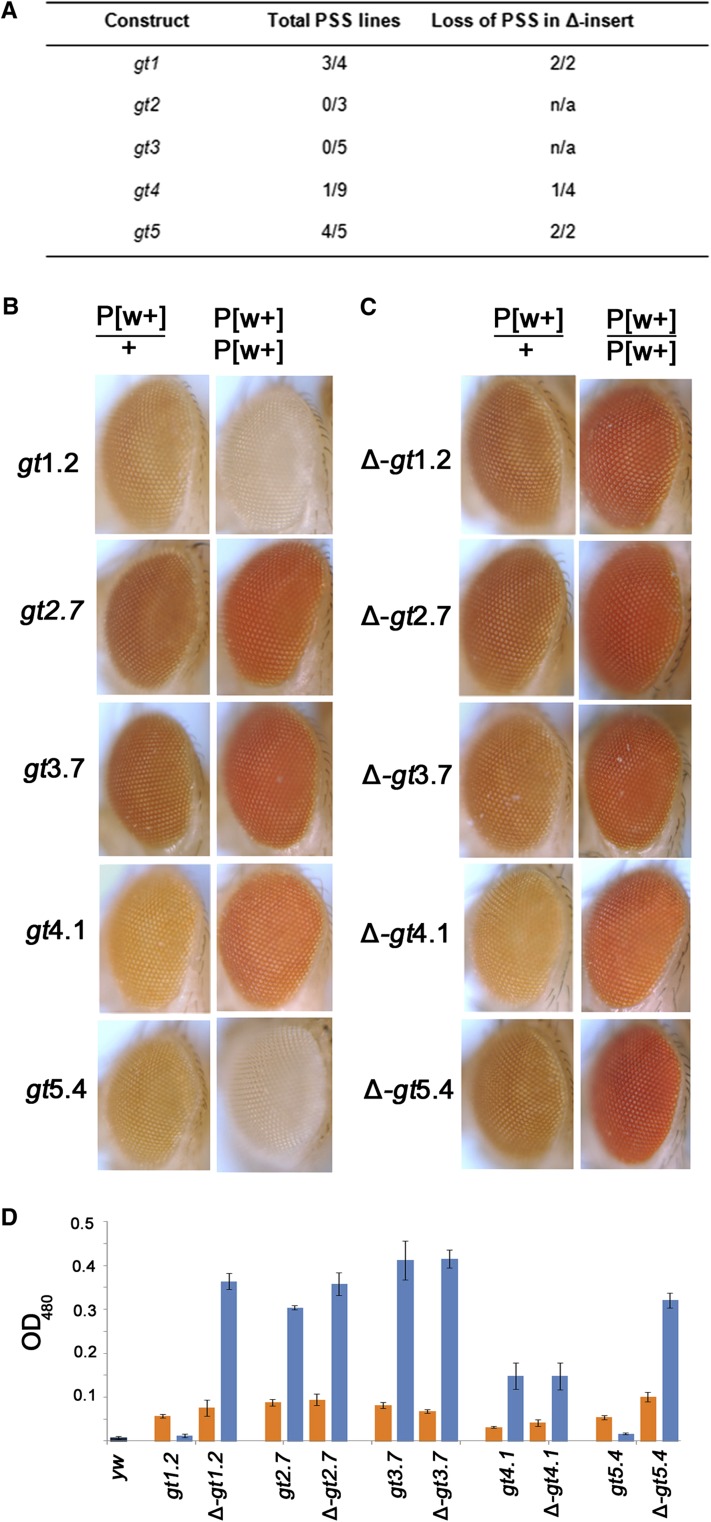
The *gt* fragments exhibit pairing-sensitive silencing. (A) The number of transgenic lines exhibiting pairing-sensitive silencing (PSS) relative to total number of lines tested. The middle column lists the results for all lines tested for each construct. The right column lists the results for the subset of lines for which deletion-derivatives were generated. These are the subset of lines that were tested for β-galactosidase expression (Table 1) and that were homozygous-viable. (B) Eye pigmentation of SD10-*gt* transgenic flies. Specific transgenic lines are indicated to the left. Flies were either heterozygous (P[w+]/+) or homozygous (P[w+]/P[w+]) for the transgene. SD10-*gt*1 and SD10-*gt*5 homozygotes showed reduced eye pigmentation compared to heterozygotes (PSS). None of the SD10-*gt*2 or SD10-*gt*3 lines exhibited pairing-sensitive silencing. Eight of nine SD10-*gt*4 lines did not exhibit PSS. (C) Deletion lines corresponding to the same lines in (B) after excision of the *gt* fragment by FLP recombinase. PSS seen in the SD10-*gt*1.2 and SD10-*gt*5.4 homozygotes was lost upon deletion of the *gt* fragment. (D) Quantitative assays of fly eye pigments of the same lines shown in (B) and (C). *yw* is the *y w^67c23^* stock that contains the transgenes. The absorbance values for heterozygotes and homozygotes of each transgene are, respectively, illustrated as orange and blue bars. Error bars represent SD. There was a statistically significant difference between absorbance values for heterozygotes and homozygotes of each line. *gt*4.1 and Δ*gt4.1*, *P* < 0.05. All other lines, *P* < 0.001.

Because some, but not all, of the previously tested PREs exhibit pairing-sensitive silencing (Kassis 2002), all SD10-*gt* transgenes that were able to homozygose were also tested for their ability to demonstrate pairing-sensitive silencing of the mini-*white* gene in the SD10 vector (Figure 4). The eye colors of transgenic flies homozygous for the transgene were compared to those of the heterozygotes. If a transgene exhibits pairing-sensitive silencing, then homozygotes will show stronger repression of the mini-*white* gene within the SD10 vector, and as a result will have lighter eye color when compared to heterozygous flies with only one copy of the transgene. Consistent with the presence or lack of PREs based on the PRE maintenance assay (Figure 2C), three out of four SD10-*gt1* and four out of five SD10-*gt5* lines demonstrated pairing-sensitive silencing compared to zero out of three SD10-*gt*2 and zero out of five SD10-*gt3* lines (Figure 4A). Curiously, although the *gt*4 fragment overlaps with *gt*5 and behaves as a PRE in the maintenance assay, only one out of nine SD10-*gt4* lines exhibited pairing-sensitive silencing (Figure 4A). Of the transgenic lines for which we have both intact transgenes and their FLP recombinase-induced derivatives (Figure 4A, right column), the results were consistent. Of this subset of lines, two out of two SD10-*gt1* lines and two out of two SD10-*gt5* lines lost pairing-sensitive silencing on deletion of the *gt* fragment. The eyes of flies from representative lines are shown in Figure 2, B and C. Deletion of the *gt* fragment from the sole SD10-*gt4* line that produced pairing-sensitive silencing also eliminated this activity (data not shown). This suggests that the region shared by *gt*4 and *gt*5 is necessary, but not sufficient, for pairing-sensitive silencing, and that additional sequences contained within *gt*5, but not *gt*4, are needed. Quantitative assays performed with the same fly lines shown in Figure 4, B and C showed eye pigment levels that were consistent with the visual appearance of eye colors (Figure 4D).

## Discussion

We have identified and mapped the locations of two PRE-containing regions at the *gt* locus. One is near the promoter and a second is ∼6 kb upstream. This organization is most similar to the PRE distribution at the *invected* (*inv*) gene, which has two PREs located at similar positions relative to the *inv* transcription start site (Cunningham *et al.* 2010). *inv* is part of a gene complex that also includes the *engrailed* (*en*) gene. *en* has two closely linked PREs that are located in an interval spanning ∼0.4 to 2.4 kb upstream of the transcription start site (Americo *et al.* 2002; Devido *et al.* 2008). The *even skipped* (*eve*) gene also has two PREs; one is near the promoter and the second PRE is ∼9 kb downstream (Fujioka *et al.* 2008). It has been suggested that multiple PREs at *inv*, *en*, and *eve* may contribute to physical interactions between remote *cis*-regulatory regions and promoters (Devido *et al.* 2008; Fujioka *et al.* 2008; Cunningham *et al.* 2010). It is likely that this activity may be responsible for the pairing-sensitive silencing observed in the context of transgenes. It is not clear to what extent multiple PREs may be functionally redundant at their endogenous loci. Each is defined by its independent ability to maintain transcriptional repression of a transgenic reporter. However, the observation that more robust repression is produced by a combination of both *en* PREs than by either PRE alone suggests that they are only partially redundant (Devido *et al.* 2008). The degree to which *gt* PREs may be functionally redundant remains to be determined. It is also possible that *gt* PREs may independently regulate distinct enhancers or may somehow cooperate in the establishment and/or maintenance of PcG-mediated repression.

Even though most PREs that test positive in maintenance assays also show pairing-sensitive silencing, it has been shown that pairing-sensitive silencing does not always correlate with PRE function. In some cases, a pairing-silencing element, conferring pairing-sensitive silencing, is distinct from a PRE. One example is the *Mcp* PRE, which regulates the *Abdominal-B* gene of the *Bithorax complex*. The 800-bp *Mcp* core fragment behaves as a functional PRE in reporter maintenance assays but is unable to demonstrate pairing-sensitive silencing unless supplemented with additional flanking sequences or possibly other regulatory sequences from other genes (Busturia *et al.* 1997; Muller *et al.* 1999). The sequence shared by *gt4* and *gt5* may be similar to the *Mcp* core fragment in that both SD10-*gt4* and SD10-*gt5* maintain repression of *en* enhancer-driven reporter expression, but only SD10-*gt5* exhibits pairing-sensitive silencing. It may be that sequences contained in *gt5*, but not *gt4*, include binding sites for proteins needed for pairing-sensitive silencing, but not for maintenance assays. Definition of the sequence requirements for pairing-sensitive silencing will require further dissection of this portion of the *gt* regulatory region and mutational analysis of potential transcription factor binding sites.

Although attempts have been made to identify PREs based on their sequences (Ringrose *et al.* 2003; Fielder and Rehmsmeier 2006), recent studies have shown that there are shortcomings to using consensus binding sites to predict the location of PREs (Schwartz *et al.* 2006; Oktaba *et al.* 2008; Cunningham *et al.* 2010). This is partially attributable to the sequence heterogeneity of characterized PREs, which reflects the variable assortment of proteins that bind to any given PRE. With the exception of Pho, only a subset of identified PRE binding proteins appears to bind to any particular PRE. Furthermore, the binding sites for several of these proteins are highly variable. For example, Pho consensus binding sites do not always correlate to Pho binding or PRE function. The core of the Pho consensus sequence was originally defined as GCCAT (Fritsch *et al.* 1999). On the basis of genome-wide ChIP-on-chip studies, an expanded consensus sequence has been proposed, G(C/A)(C/G)GCCAT(T/C)TT (Oktaba *et al.* 2008). Even more perplexing, Pho has been shown to bind to fragments that contain neither of these consensus sequences (Cunningham *et al.* 2010).

**Figure 5:**
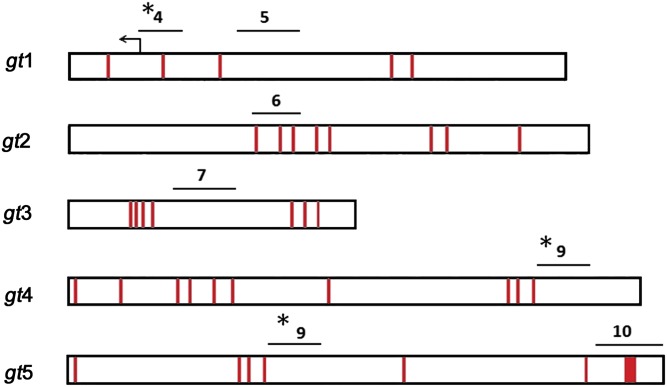
Locations of Pho consensus binding sites within *gt* fragments. Schematic of *gt* fragments with Pho core consensus sites (GCCAT) in red. Lines above each *gt* fragment represent regions amplified by PCR in ChIP assays. Pho-positive regions (see Figure 1B) are indicated with asterisks.

Examination of the *gt1*, *gt4*, and *gt5* sequences reveals that, although Pho consensus sites coincide with the ChIP-defined localization of Pho in embryos (Figure 1B), Pho does not appear to bind to regions that contain many additional Pho consensus sites (Figure 5 and Table 2). For example, the *gt*2 and *gt3* fragments have eight and seven Pho consensus sites, respectively, but are negative for Pho binding in ChIP assays and fail to behave as PREs in the maintenance and pairing-sensitive silencing assays. The extended Pho-consensus sequence (Oktaba *et al.* 2008) is not present within the *gt* regulatory region. It seems probable that a PRE is more likely to be determined by the presence of multiple binding sites for an array of DNA-binding proteins, and that Pho binding may involve cooperative interactions with some combination of other DNA binding factors (Kassis and Brown 2013). It is also possible that Pho may be bound to other regions in the *gt cis*-regulatory region at later developmental stages.

**Table 2:** Summary of Pho consensus sites within *gt* fragments

**Fragment**	**Coordinates**	**Pho Consensus Sites**
*gt*1	+274 to −1629	5
*gt*2	−1143 to −3152	8
*gt1*, *2* overlap	−1143 to −1629	0
*gt3*	−3032 to −4150	7
*gt4*	−4302 to −6539	10
*gt5*	−5341 to −7641	10
*gt4*, *5* overlap	−5341 to −6539	4

Summary of Pho consensus sites within *gt* fragments. Coordinates of *gt* fragments relative to the transcription start site and summary of Pho consensus sites within each fragment. *gt*1 and *gt*2 overlap by 486 bp. *gt*4 and *gt*5 overlap by 1198 bp.

In conclusion, we have demonstrated the presence of two *gt* PRE-containing regions within the *gt cis*-regulatory region. Consistent with other characterized PREs, both *gt* PRE-containing regions correspond to localized binding by Pho *in vivo*. The presence of Pho at a single location within each of these regions indicates that it is likely that each contains just one PRE. However, at this time we cannot rule out the possibility that one or both regions may contain multiple closely linked PREs.

## Supplementary Material

Supporting Information
