# Supplementary material for: Mapping Polycomb Response Elements at the Drosophila melanogaster giant Locus
Source: G3 (Bethesda). 2013 Oct 29;3(12):2297–304. doi: 10.1534/g3.113.008896 (PMC3852391; doi:10.1534/g3.113.008896)
Supplement: Supporting Information [file supp_3_12_2297__index.html]

Mapping Polycomb Response Elements at the Drosophilla melanogaster giant Locus — Supporting Information 

# Mapping Polycomb Response Elements at the *Drosophila melanogaster giant* Locus

## Supporting Information for AlHaj Abed *et al.*, 2013

**Files in this Data Supplement:**

- Supporting Information - Tables S1-S2 (PDF, 427 KB)
- Table S1 - Cloning primers (PDF, 294 KB)
- Table S2 - Primers used for ChIP (PDF, 296 KB)
