# Supplementary material for: Mapping Polycomb Response Elements at the Drosophila melanogaster giant Locus
Source: G3 (Bethesda). 2013 Oct 29;3(12):2297–304. doi: 10.1534/g3.113.008896 (PMC3852391; doi:10.1534/g3.113.008896)
Supplement: Supporting Information [file supp_g3.113.008896_TableS1.pdf]

Table S1 Cloning primers

Primer	Sequence
gt1-U	5’- GGCGACATGTGAAGTTCCTATACTTTCTAGAGAATAGGAACTTCTGCTGGTACTGGTGGTGGTG -3’
gt1-L	5’- GGCGACATGTGAAGTTCCTATTCTCTAGAAAGTATAGGAACTTCGAATGGGCAGTTGTAAAAAGAG -3’
gt2-U	5’- GGGACATGTGAAGTTCCTATACTTTCTAGAGAATAGGAACTTCTCGCATCCTTTTCTTATTTCG -3’
gt2-L	5’- GGGACATGTGAAGTTCCTATTCTCTAGAAAGTATAGGAACTTCTACCTGTTCCCATTATCCTCTG -3’
gt3-U	5’- GCCACATGTGAAGTTCCTATACTTTCTAGAGAATAGGAACTTCAGCGAAAAAGACTAACCAGGA -3’
gt3-L	5’- GCCACATGTGAAGTTCCTATTCTCTAGAAAGTATAGGAACTTCGCAGTGTAGCCGTAGGAGCA -3’
gt4-U	5’- GCCACATGTGAAGTTCCTATACTTTCTAGAGAATAGGAACTTCCATTCGTTTTGATGAACTGTCA -3’
gt4-L	5’- GCCACATGTGAAGTTCCTATTCTCTAGAAAGTATAGGAACTTCATGCCCCCGATGCTGTCT -3’
gt5-U	5’- CGCACATGTGAAGTTCCTATACTTTCTAGAGAATAGGAACTTCGCACGCAGTTTTGTAGGTGG -3’
gt5-L	5’- CGCACATGTGAAGTTCCTATTCTCTAGAAAGTATATAGGAACTTCGCGGTCCTCAGTTCTCAGTC -3’
