# Supplementary material for: Mapping Polycomb Response Elements at the Drosophila melanogaster giant Locus
Source: G3 (Bethesda). 2013 Oct 29;3(12):2297–304. doi: 10.1534/g3.113.008896 (PMC3852391; doi:10.1534/g3.113.008896)
Supplement: Supporting Information [file supp_g3.113.008896_TableS2.pdf]

Table S2   Primers used for ChIP

Primer	Sequence	Length of amplicon	Coordinate from TSS
1	5'- TCCGTTTTTCTGCGCTTGTAG -3'	214	+4048
	5'- GGATCTGAATATGAATGTGGAGGTT -3		+3834
2	5' TGAAATTCATTCCTTAGGGCTAT -3'	218	+1834
	5' AGGGGGCTCAACTTTCTTTT -3'		+1616
4	5' AACGCAAACTGATTTCCTCTCG -3'	177	+9
	5' CAGAAGCAAAGCCAGAATCACC 3'		-168
5	5'- TTCCGCGAAAGGATATGGTT -3'	239	-368
	5'- AAATCAAATGCACAATGCTGGT -3'		-607
6	5' -CGT ATA GCC CAG CCC AATC -3'	182	-1839
	5' -GCT CAT TAT GGC GAA GGA ACA -3'		-2021
7	5' -CTG ACC AGC CAA GCG AAA AG-3'	240	-3422
	5' - GGC CGG TGC AAA CTT AAG ATA G - 3'		-3662
9	5' -CCG GGC CAT GCA ATA AAG TA -3'	212	-6108
	5' -CGC TTC CTC CAA CTC CCT ATA TTC-3'		-6320
10	5' -ATA TGC CAC GCC ATC TTA GCA C -3'	258	-7378
	5' -CCT CAG TTC TCA GTC CGC TTC TAA T -3'		-7636
13	5'- CCAAATGCCACACACAACACA -3'	233	-14263
	5'- GCCAGTTTCACATGCACATCAA -3'		-14496
